# Experimental Infection of Dogs with Avian-Origin Canine Influenza A Virus (H3N2)

**DOI:** 10.3201/eid1501.080755

**Published:** 2009-01

**Authors:** Daesub Song, Chulseung Lee, Bokyu Kang, Kwonil Jung, Taehoon Oh, Hyekwon Kim, Bongkyun Park, Jinsik Oh

**Affiliations:** Green Cross Veterinary Products, Yong-in, South Korea (D. Song, C. Lee, B. Kang, T. Oh); Daewoong Pharmaceutical Company, Kyounggi-Do, South Korea (K. Jung); Seoul National University, Seoul, South Korea (H. Kim, B. Park); Animal Genetics, Inc., Suwon, South Korea (J. Oh); 1 These authors contributed equally to this article.

**Keywords:** contact infection, canine, influenza virus, dispatch

## Abstract

Susceptible dogs were brought into contact with dogs experimentally infected with an avian-origin influenza A virus (H3N2) that had been isolated from a pet dog with severe respiratory syndrome. All the experimentally infected and contact-exposed dogs showed elevated rectal temperatures, virus shedding, seroconversion, and severe necrotizing tracheobronchitis and bronchioalveolitis.

Transmission of highly pathogenic avian-origin canine influenza A viruses (H3N2) that spread across South Korea during May through December 2007 was observed repeatedly in the country’s animal clinics (*1*). These viruses share >97% nucleotide sequence homology, suggesting that whole viruses were transmitted directly from birds to dogs. To determine whether these viruses can be transmitted directly from dog to dog, we experimentally infected beagles by direct contact. Dog-to-dog transmission of the virus raises questions about the interspecies transmission of avian influenza viruses and adaptation of these viruses to canine physiology.

## The Study

Dogs in the study comprised 3 groups of beagles housed in different rooms of the isolation facility at Green Cross Veterinary Products (Yong-in, South Korea). The virus used was avian-origin canine influenza virus A/canine/01/2007, subtype H3N2, which had been isolated from a pet dog with severe respiratory syndrome. In the first group (challenge group), 4 beagles were inoculated intranasally with a 10^6.5^ 50% egg infectious dose (EID_50_). Two hours later, the second group of 4 uninfected dogs (exposure group) was housed in the same contaminant room. These uninoculated dogs had frequent direct nose-to-nose contact with the inoculated dogs. The third group of 4 dogs (control group) was housed separately as uninoculated controls. Rectal temperatures were checked and nasal swab samples were collected daily. We monitored clinical signs of infection 7 days postinoculation (dpi) and examined nasal swabs obtained 10 dpi for virus shedding; serum samples were collected at 0, 3, 7, 9, and 13 dpi. Serum antibodies against nucleoprotein were detected by using a commercial competitive ELISA (Animal Genetics, Inc., Suwon, South Korea). On 7 and 13 dpi, 2 dogs from each group were euthanized for gross and histopathologic examination. All organs from the dogs were rapidly immersed in 10% neutral formalin buffer to prevent autolysis and stored overnight. All animal experiments complied with the current laws of South Korea. Animal care and treatment were conducted in accordance with guidelines established by the Seoul National University Institutional Animal Care and Use Committee. A p value <0.01 was considered statistically significant.

**Figure 1:**
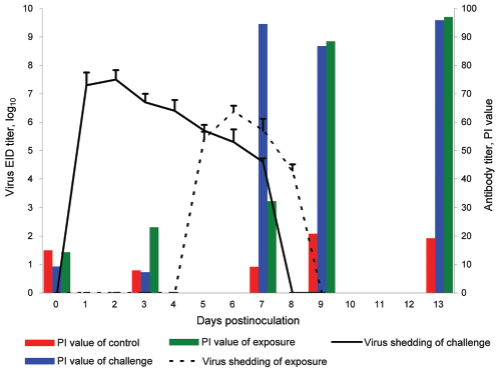
Histopathologic appearance of tissue of dogs experimentally exposed to canine influenza virus by contact with infected dogs. Severe necrotizing, suppurative tracheitis and bronchioalveolitis were observed in the contact-exposure group on day postinoculation (dpi) 13. However, influenza-associated lesions were not yet present in these dogs on dpi 7. Original magnification was ×200 for all images. Hematoxylin and eosin stain.

**Figure 2:**
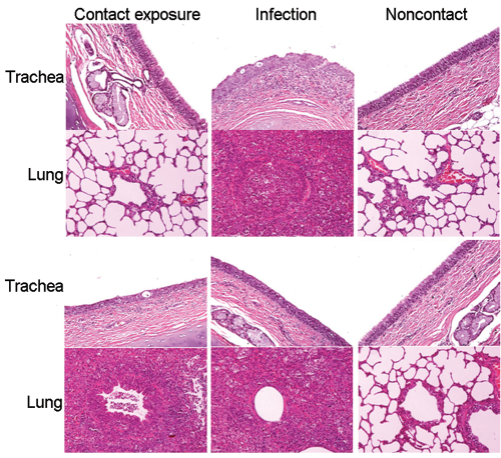
Virus shedding and serologic response of beagles after experimental contact transmission of canine influenza virus. EID, egg infectious dose; PI, percentage inhibition.

Clinical signs, including sneezing, nasal discharge, and coughing, were observed 2–8 dpi in the challenge group and 5–8 dpi in the exposure group. Twenty-four hours after inoculation, fever developed in dogs in the challenge group (mean rectal temperature 39.85°C–39.75°C) that lasted until 3 dpi. Fever (39.5°C) developed in dogs in the exposure group 72 hours after exposure; mean rectal temperature was 38.65°C a day later. Fever and clinical signs were not observed in the control group. Necropsy examination found gross lesions in the lung, including multifocal to coalescing reddish consolidations, consistent with influenza-induced lung lesions in other species. These gross lesions were observed in the challenge group on dpi 7 and 13 but in the exposure group only on dpi 13. Histopathologic examination showed severe necrosis and inflammation of the upper and lower respiratory tracts. Infected dogs shared the following histopathologic features (Figure 1): 1) severe multilobular or diffuse necrotizing tracheobronchitis with suppurative inflammation in the lumina and squamous metaplasia of the tracheobronchial epithelium and 2) severe multilobular bronchiolitis and alveolitis. We observed these histopathologic lesions in the trachea and lungs in the challenge group on dpi 7 and 13 but in the exposure group only on dpi 13. For the challenge group, mean virus titers in respiratory tract tissues were 10^3.9^ EID_50_/mL on dpi 7, but no viruses were detected in the tissues on dpi 13. For the contact group, mean virus titers in respiratory tract tissues were 10^4.7^ EID_50_/mL on dpi 7, but no viruses were detected in the tissues on dpi 13. We detected viruses in nasal discharge of dogs in the challenge group on dpi 2–6 and in the exposure group on dpi 5–8 (p<0.01). Shedding peaked on dpi 2 in the challenge group and dpi 6 in the exposure group (Figure 2). Nucleoprotein-specific ELISAs showed that all dogs lacked nucleoprotein-specific antibodies before inoculation and that dogs in the control group remained negative throughout the experiment. However, the percentage inhibition values were positive on dpi 7, 9, and 13 for dogs in the challenge group (p<0.01) and positive on dpi 9 and 13 for dogs in the exposure group (p<0.01, cut-off ratio of percentage inhibition >50) (Figure 2).

Transmission of virus from 1 host to another species—in general, essentially unaltered by direct transfer—is an important feature of the ecology of influenza virus (*2*). Examples of this interspecies transmission mechanism include the recent human infections with the H5N1 subtype of avian influenza virus (*3*,*4*). Dogs infected with avian subtype H3N2 recently were identified in South Korea, suggesting that an avian influenza virus with high pathogenicity that can rapidly spread from dogs to dogs has made the interspecies leap**.** Most whole influenza viruses that are transmitted directly from the natural host species to a different species do not achieve sustained transmission in the new host species (*5*), suggesting that multiple virus–host interactions are needed before the virus can replicate and be transmitted horizontally in a new host species (*2*). Here we showed that close contact between canine influenza virus-infected and –noninfected dogs results in spread of the virus to the uninfected dogs, which then develop clinical signs of the disease.

## Conclusions

We showed that an avian-origin canine influenza virus isolated from a pet dog can spread from dog to dog by contact infection. We observed a transient rise in rectal temperature in the challenge and exposure dogs and seroconversion in the exposure dogs. These dogs also had viral RNA in their nasal swabs and histopathologic changes in their upper and lower respiratory tracts. Our results demonstrate that an avian-origin canine influenza virus readily infects dogs and is easily transmissible among dogs.
